# Developing methods to study conformational changes in RNA crystals using a photocaged ligand

**DOI:** 10.3389/fmolb.2022.964595

**Published:** 2022-08-16

**Authors:** Hyun Kyung Lee, Chelsie E. Conrad, Valentin Magidson, William F. Heinz, Gary Pauly, Ping Yu, Saminathan Ramakrishnan, Jason R. Stagno, Yun-Xing Wang

**Affiliations:** ^1^ Protein-Nucleic Acid Interaction Section, Center for Structural Biology, Center for Cancer Research, National Cancer Institute, Frederick, MD, United States; ^2^ Optical Microscopy and Analysis Laboratory, Cancer Research Technology Program, Frederick National Laboratory for Cancer Research, Frederick, MD, United States; ^3^ Chemical Biology Laboratory, Center for Cancer Research, National Cancer Institute, Frederick, MD, United States

**Keywords:** crystal phase transition, photocaged ligand, photo-induced, RNA, riboswitch

## Abstract

Crystallographic observation of structural changes in real time requires that those changes be uniform both spatially and temporally. A primary challenge with time-resolved ligand-mixing diffraction experiments is asynchrony caused by variable factors, such as efficiency of mixing, rate of diffusion, crystal size, and subsequently, conformational heterogeneity. One method of minimizing such variability is use of a photolabile caged ligand, which can fully saturate the crystal environment (spatially), and whose photoactivation can rapidly (temporally) trigger the reaction in a controlled manner. Our recently published results on a ligand-mixing experiment using time-resolved X-ray crystallography (TRX) with an X-ray free electron laser (XFEL) demonstrated that large conformational changes upon ligand binding resulted in a solid-to-solid phase transition (SSPT), while maintaining Bragg diffraction. Here we investigate this SSPT by polarized video microscopy (PVM) after light-triggered release of a photo-caged adenine (pcADE). In general, the mean transition times and transition widths of the SSPT were less dependent on crystal size than what was observed in previous PVM studies with direct ADE mixing. Instead, the photo-induced transition appears to be heavily influenced by the equilibrium between caged and uncaged ADE due to relatively low sample exposure and uncaging efficiency. Nevertheless, we successfully demonstrate a method for the characterization of phase transitions in RNA crystals that are inducible with a photocaged ligand. The transition data for three crystals of different sizes were then applied to kinetic analysis by fitting to the known four-state model associated with ligand-induced conformational changes, revealing an apparent concentration of uncaged ADE in crystal of 0.43–0.46 mM. These results provide further insight into approaches to study time-resolved ligand-induced conformational changes in crystals, and in particular, highlight the feasibility of triggering phase transitions using a light-inducible system. Developing such approaches may be paramount for the rapidly emerging field of time-resolved crystallography.

## 1 Introduction

Riboswitches are highly structured RNA elements found in the untranslated regions of some mRNAs that confer regulatory control over transcription or translation through the binding of cellular metabolites involved in several biosynthetic pathways. A typical riboswitch contains two domains. The aptamer (ligand-sensing) domain undergoes conformational changes in response to ligand binding. These structural rearrangements alter the stability of a mutually exclusive secondary structural element in the expression platform (regulatory domain) resulting in a ligand-controlled ON/OFF switch (Garst et al., 2011). Adenine riboswitches, as indicated by the name, regulate gene expression in response to adenine and are among the purine family of riboswitches, which includes adenine, guanine, and 2′-deoxyguanosine. These riboswitches share many similarities in both sequence and biochemistry, and have comparable secondary and tertiary structures (Mandal and Breaker, 2004). The *add* adenine riboswitch (riboA) in particular has served as a model system for investigating ligand-induced conformational changes in the aptamer domain and providing a structural basis for how these changes are associated with gene regulation. Although significant progress has been made on this front, we seek to derive a molecular movie that maps out these conformational changes through elucidation of transient structures along the trajectory using time-resolved X-ray crystallography (TRX) with an XFEL.

TRX has advanced significantly with the development of XFELs, whose micro-focused X-ray pulses with enormous intensities enable room-temperature crystallography experiments using a continuous stream of micron-sized crystals that can be manipulated and probed in real time. The femtosecond pulse durations allow for “diffraction-before-destruction,” that is the diffraction of each crystal outruns the radiation damage that then immediately vaporizes the sample (Neutze et al., 2000; Liu and Lee, 2019; Brändén and Neutze, 2021). Using this approach, scientists have been able to observe macromolecular changes that were otherwise unattainable. Light-sensitive proteins and photosystems (e.g., photosystem II, photoactive yellow protein, phytochromes, and reversibly switchable fluorescent proteins) have been studied extensively for their changes in structure throughout their photocycles (Kupitz et al., 2014; Tenboer et al., 2014; Young et al., 2016; Suga et al., 2019; Claesson et al., 2020; Ibrahim et al., 2020; Orville, 2020; Woodhouse et al., 2020). However, such TRX experiments become more difficult with ligand-inducible reactions that involve much more substantial conformational changes. Here, the method of mix-and-inject serial crystallography is used in which the sample and ligand are mixed at defined and tunable time intervals prior to entering the X-ray interaction region (Stagno et al., 2017a; Stagno et al., 2017b; Kupitz et al., 2017; Ji-Hye et al., 2018; Schmidt, 2020; Ramakrishnan et al., 2021a). Using this approach with riboA crystals, we determined distinct conformational states of the aptamer, including a ligand-bound intermediate. For the first time, it was demonstrated that TRX was capable of structure elucidation of a riboswitch during its conversion to the ligand-bound state (Stagno et al., 2017b; Ramakrishnan et al., 2021a). This was an important advance, not just for riboswitches, but also for opening new possibilities for studying biomolecular changes in real time crystallographically.

Due to the extensive conformational changes induced by ligand, the riboA crystals undergo the phenomenon of a solid-to-solid phase transition (SSPT), which involves a minimum of three distinct crystal lattices, from monoclinic, to triclinic, and finally to orthorhombic, and was shown to be reversible (Stagno et al., 2017b; Ramakrishnan et al., 2021a). SSPTs describe the transformation of a crystalline solid without going through the liquid phase, and are commonly observed in many organic and inorganic crystals. Examples of SSPTs range from different forms of water-ice at different pressures (Tae, 2019), the transition of graphite to diamond (Cheng, 2008), hysteresis in electronic crystals (Lv et al., 2022), to phase changes of shape memory alloys (Otsuka and Wayman, 1999). SSPTs are less common in biomolecular crystals but have been reported, e.g., aliphatic linear-chain amino acid crystals (Smets et al., 2020) and lysozyme (Berthou and Jolles, 1974). However, SSPTs described for protein crystals typically do not involve major conformational changes, as were observed in riboA crystals. We previously developed a method using polarized video microscopy (PVM) to characterize the ligand-induced SSPT in riboA crystals by monitoring transmitted intensity changes in the crystal under cross-polarized light, and also using these data to simulate the associated reaction kinetics (Ramakrishnan et al., 2021a; Ramakrishnan et al., 2021b; Ramakrishnan et al., 2021c; Ramakrishnan et al., 2021d) For such a transition to occur and maintain lattice order, the displacive transformation from one structure to the other must be spatiotemporally cooperative, which becomes increasingly difficult with larger molecules, larger conformational changes, and more complex lattice interactions. The ability of macromolecular crystals to undergo such drastic changes depends in part on their size, where smaller crystals may benefit from having higher surface-to-volume ratios that enable more rapid diffusion and a greater degree of plasticity to accommodate changing lattice environments (Schmidt, 2013; Ramakrishnan et al., 2021d). To this end, we have employed TRX with an XFEL on riboA crystals upon mixing with ligand (Ramakrishnan et al., 2021a). Although key structural information could be derived from these studies, the diffraction data were convoluted, and extracting dynamic structural information was fraught with challenges in lattice interpretation and sorting of reflection intensities. We postulated that the SSPT triggers only when enough energy has accumulated in the form of the ligand-bound intermediate. Therefore, despite the fact that diffusion rates are expected to be rapid relative to the detectable onset of the SSPT (seconds), the gradual and non-uniform molecular perturbations induced by ligand interactions during this rate-limiting step may explain some of the non-uniformity in transition. In this study, we investigate the SSPT in riboA crystals using a photocaged ligand and a light-inducible mechanism as a potential means to achieve greater uniformity of transition for diffraction studies.

## 2 Materials and methods

### 2.1 Solution preparation

Crystallization buffer consisted of 40 mM sodium cacodylate pH 6.5, 100 mM MgCl_2_, 12 mM spermine tetrahydrochloride, and 28%–38% (v/v) 2-methyl-2,4-pentanediol (MPD). RNA buffer consisted of 10 mM HEPES pH 7.5, 100 mM KCl, and 0.5 mM EDTA. The stabilization buffer was made by mixing crystallization buffer (containing 65% MPD) with RNA buffer in a 1:1 ratio. The solutions were kept at room temperature. The photocaged adenine (pcADE) solution was prepared at 10 mM by adding 3 mg of pcADE to 1 ml of stabilization buffer in a tube impervious to light, followed by heating at 95°C for 2 min, and then vortexing. The process of heating and vortexing was repeated until a total of 10 min of heating was reached, or until the compound was completely dissolved. The pcADE solution was freshly prepared each day and kept in a water bath at 45°C to prevent precipitation. The organic synthesis and photolytic reaction of pcADE has been described elsewhere (Supplementary Figure S1) (Ramakrishnan et al., 2021a).

### 2.2 Adenine riboswitch crystallization

The crystals used for PVM experiments were grown using the sitting-drop vapor-diffusion method. 500 µl of crystallization buffer ranging from 28% to 38% MPD was dispensed into each well. Then, 2.5 µl of 7.5 g/L native-gel-purified riboA was mixed with 2.5 µl of crystallization buffer, and the tray was sealed with tape. After letting the drop equilibrate for 90 min, 0.5 µl of crystal seed stock ranging from 1:10 to 1:10,000 dilution was gently added to the drop without mixing. The tray was then resealed with tape and incubated overnight at 22°C.

### 2.3 Experimental setup and time-lapse polarized video microscopy recording

From the crystallization drop, 1 µl of crystals was harvested and diluted with 9 µl of 10 mM pcADE solution. The crystals were soaked for at least 3 min to ensure complete saturation with ligand. 1 µl of the pcADE-soaked crystals was then placed onto a poly-d-lysine-coated glass-bottom dish. The poly-d-lysine helped prevent excessive motion of crystals during the SSPT. In order to prevent the drop from drying out and to help pcADE stay in solution, a circle of vacuum grease was placed around the drop to create a barrier, and 30 µl of stabilization buffer were added outside of the grease. The well of the dish was then covered with a glass coverslip to further prevent evaporation. PVM imaging was done using a Nikon Eclipse Ti2-E inverted microscope with a x100/1.45 Plan Apo Lambda Oil objective (UV passable), a 0.85 HNA dry condenser lens, a filter cube with a 365-nm DAPI dichroic mirror (Nikon), a polarizer below the objective (Nikon TI2-C-DICA), and a polarizer on a motorized precision rotation stage (Thorlabs KPRM1E). The riboA crystals were centered and focused in the microscope field of view, and the intensity of the transmitted light through the birefringent crystals was optimized by adjusting the angle of cross-polarization. Epi-illumination through the microscope objective was performed using a SOLIS-365C UV LED, mounted on the back port of the microscope, powered by a DC2200 LED driver (Thorlabs). The crystals were flashed for 5 s at 365 nm with an LED current of 4.5 Amps. Videos were recorded from the time of UV exposure (*t* = 0) to 6–8 min thereafter.

### 2.4 Video processing and analysis

Time-lapse PVM data were visualized and processed using ImageJ (Schneider et al., 2012) and analyzed using custom MATLAB scripts (*OMAL_PVM_Analyzer*), as described previously (Ramakrishnan et al., 2021c). Briefly, the raw videos were exported as .AVI files and then imported into ImageJ as 8-bit virtual stacks in grayscale. Regions of interest (ROIs) were cropped, saved as new .AVI files, and then processed with *OMAL_PVM_Analyzer* using MATLAB (v. 2019b, MathWorks) to generate intensity plots and their first-derivative curves. Square neighborhoods of pixels ranging in length from 1 to 2 pixels were used to calculate average intensities for spatially binned superpixels, and were smoothed using a boxcar average window of 65 and 39 for positional variance and ROI size analyses, respectively. The times of transition (Τ1) and their durations were derived from the primary peaks in the first derivative curves, and were further classified using MATLAB’s *k-means* clustering algorithm (Lloyd, 1982; Arthur and Vassilvitskii, 2007). Results of the PVM analysis were written as .CSV files and the data were normalized and graphed using GraphPad Prism 9.

### 2.5 Uncaging efficiency

#### 2.5.1 HPLC analysis

The percentage of ADE released upon UV photolysis was quantified using an experimental setup mirroring the PVM experiments with riboA crystals. A 1 µl drop of pcADE solution at a measured concentration was placed onto a glass-bottom dish and illuminated with the UV LED for durations of 1, 5, or 10 s. The drop was immediately harvested with stabilization buffer (1:50 dilution) and the products were separated and quantified by analytical HPLC (Varian Prostar 210) using a Sunfire C18 reverse-phased column (4.6 mm × 150 mm, 3.5 µm pore size; flow rate 0.5 ml/min), with an elution gradient as follows: equilibration and sample injection at 100% Solvent A, 0%–100% Solvent B over 25 min, holding at 100% Solvent B for an additional 20 min, then re-equilibration at 100% Solvent A for 3 min. Solvent A = 20 mM sodium phosphate pH 6; Solvent B = 20 mM sodium phosphate pH 6, 50% (v/v) acetonitrile. The column was washed 10 min with 100% acetonitrile, followed by 30 min with Solvent A between each run.

#### 2.5.2 Standard curve for adenine quantification

Theoretically, the loss in pcADE should equal the gain in ADE product. In practice, however, we found that this wasn’t exactly the case, and the ratio between the two elution peaks was an inaccurate means of quantification. Instead, the production of ADE was quantified directly against established standards. A standard curve (*R* ^2^ = 0.9892) was generated for ADE by loading known quantities (∼2, 4, 6, and 8 nmol) of each (determined by UV spectrophotometry) and measuring the associated peak areas (Supplementary Figure S2). The experiments were repeated in triplicates, and the average values were determined for standardization. Then, for experimental runs, the standard curve was used to calculate the % ADE released by comparing the area of the ADE elution peak relative to the expected peak area for the mass of ADE loaded.

### 2.6 UV LED beam diameter and power measurements

The diameter of the microscope-integrated UV LED beam hitting the crystals in the PVM experiments was determined by photobleaching. A section of a cover slide was colored with a red permanent marker, and then mounted on the microscope. The colored area was exposed to the LED beam illuminating through a x100/1.45 Plan Apo Lambda oil objective for 2 min at 4.5 Amps (Supplementary Figure S3). The diameter of the bleached area was measured using Nikon NIS-Elements based on a calibration of 2.70 pixels/µm from the 20x/0.75 Plan Apo objective image, giving a minimum beam diameter of ∼0.24 mm. The diameter of a 1 µl drop (∼2.1 mm) was measured using a Zeiss Axio Zoom. V16 with a Plan-NEOFLUAR Z 1.0x/0.25 FWD 56 mm objective, based on a calibration of 0.76 pix/µm (Supplementary Figure S4).

To approximate the energy received by the sample upon UV LED exposure, a S170C microscope slide photodiode power sensor (Thorlabs) was used with a PM100D power meter (Thorlabs). The power output of the microscope-integrated UV LED after passing through the optics was measured by placing the power sensor onto the microscope stage in place of the sample and exposing it with the LED at 4.5 Amps. The power was measured to be 12.7 mW, giving a power density of ∼28 W/cm^2^, based on a beam diameter of 0.24 mm (see above and Supplementary Figure S3A). To ensure the LED beam through the objective was uniform, we measured the power at different apertures using a field stopper (Nikon TI2-F-FSC) inserted immediately upstream of the LED source (Supplementary Figure S3B). Next, the power of the LED with direct exposure was measured by mounting the LED on a vertical support, attaching a condenser lens (Thorlabs ACL25416U-A) in a lens tube (Thorlabs SM2L20), and adjusting the height above the power sensor (∼10 cm, measured from the top of the lens tube) to achieve a focused beam (∼3.25 × 3.25 mm^2^). The condenser lens was inverted in the tube for easier access to the sample. A mounted pinhole P1000HK (Thorlabs) with 1 mm aperture was inserted into black foil and placed on top of the sensor. The LED was turned on at 4.5 Amps. The pinhole position was varied horizontally to capture different parts of the beam, giving an average power reading of 120 mW, and a power density of ∼15 W/cm^2^.

### 2.7 Kinetic simulations

Simulations of the multivariance transition kinetics associated with the SSPT were performed using a custom MATLAB script and manual fitting of parameters to match the observed transitions by PVM. The data were fit based on the four-state solution kinetics model (Eq. 1) as described previously (Stagno et al., 2017b; Ramakrishnan et al., 2021a), where apo1 and apo2 are unique apo conformations, IB∙ade is a ligand-bound intermediate, B∙ade is the conformationally switched ligand-bound conformation, k_*op*_, k_*on*_, and k_*f*_ are the forward rate constants, and k_*cl*_, k_*off*_, and k_*r*_ are the reverse rate constants. As the transition in crystal is unidirectional in this case, the reverse rate constants were set to 0.

$$\mathrm{apo1}\overset{k_{\mathrm{op}}}{\underset{k_{\mathrm{cl}}}{⇌}}\mathrm{apo2+ade}\overset{k_{\mathrm{on}}}{\underset{k_{\mathrm{off}}}{⇌}}\mathrm{IB*.ade}\overset{k_{f}}{\underset{k_{r}}{⇌}}B.\mathrm{ade}$$ (1)

## 3 Results

**FIGURE 1:**
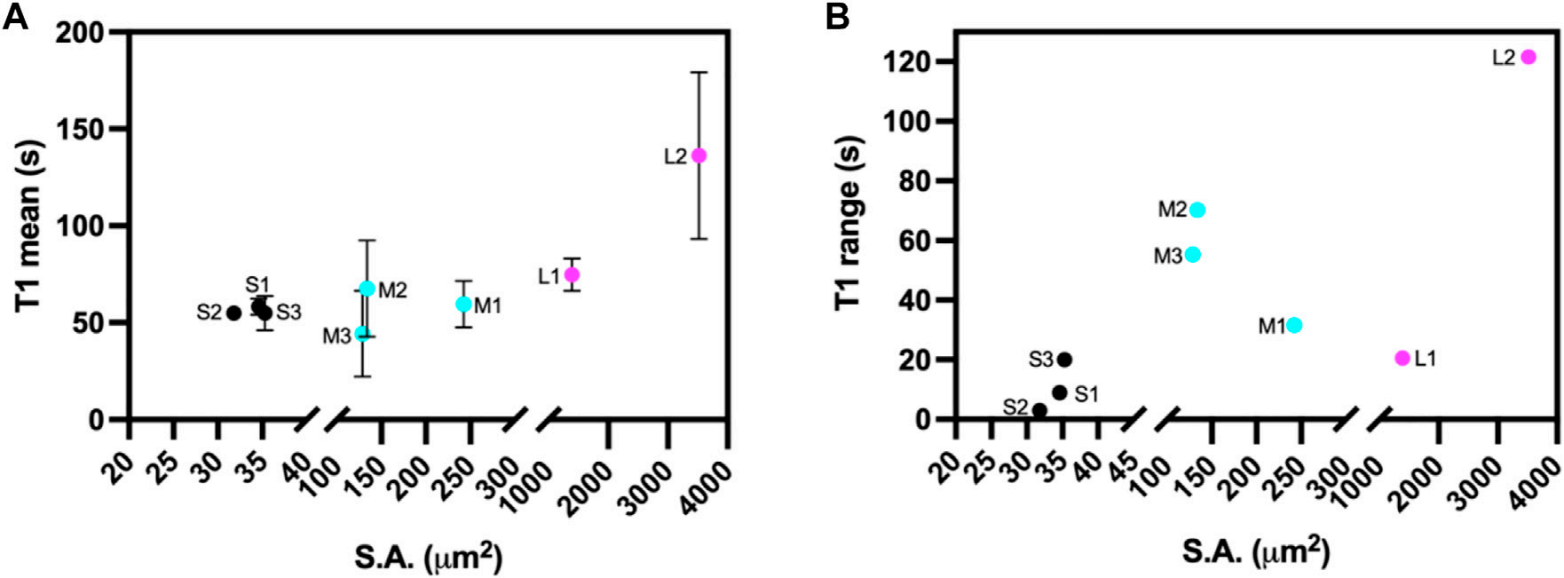
**(A)** Mean transition time (T1) and **(B)** T1 range for ROIs at different crystals positions for the eight different sized riboA crystals plotted as a function of crystal size, based on observable surface area (S.A.) Small crystals are indicated in black, medium crystals in cyan, and large crystals in magenta.

The light-induced SSPT in riboA crystals was analyzed using PVM by monitoring the changes in transmitted light intensity from birefringence. The analysis included eight crystals whose longest edge ranged from 5.4 to 53.3 µm (Supplementary Figure S5). The crystals were categorized according to their observable surface area (x-y dimensions): 31–35 μm^2^ (S1, S2, S3), 129–242 μm^2^ (M1, M2, M3), and 1,392–3,520 μm^2^ (L1, L2) (Figure 1, Table 1).

**TABLE 1:** Crystal *x*-*y* dimensions, mean transition time (T1), and transition half-width at half-max (HWHM) values for the ROIs analyzed for all eight riboA crystals.

	Dimensions (µm)	Surface Area (µm)	Mean T1 (s)	HWHM (s)
S1	5.4 × 6.4	34.6	58.3 ± 4.2	14.6 ± 2.7
S2	5.3 × 6.0	31.8	54.9 ± 1.3	17.2 ± 2.5
S3	5.7 × 6.2	35.3	54.9 ± 8.8	17.8 ± 4.6
M1	15.2 × 16.0	242.3	59.6 ± 12.0	13.0 ± 6.0
M2	8.4 × 15.9	133.9	67.6 ± 24.9	10.6 ± 1.6
M3	8.5 × 15.2	128.7	44.3 ± 22.3	9.8 ± 2.4
L1	33.0 × 42.2	1,391.7	74.8 ± 8.4	16.4 ± 3.4
L2	47.9 × 73.4	3,519.5	136.3 ± 43.1	13.7 ± 1.6

### 3.1 Efficiency of pcADE uncaging

The percent yields of UV-induced uncaging of pcADE were measured under varying experimental conditions. In each case, known quantities of caged ligand in 1 µl stabilization buffer were exposed to flashes of UV light, and the reaction products were immediately separated and quantified by HPLC analysis. The first set of experiments were performed with the LED source directly (no microscope). UV exposure times of 1, 5, and 10 s yielded peak areas corresponding to 35%, 62%, and 64% release of ADE, respectively (Table 2). To simulate the PVM experiments with riboA crystals, the LED was then integrated with the microscope optics, and the experiment was repeated, yielding 9%, 25%, and 28% release of ADE for the three exposure times (Table 2). The 2-3-fold decrease in uncaging efficiency is due to the fractional volume of liquid that is illuminated (beam diameter of 0.24 mm) relative to the entire pcADE-containing drop (drop diameter of 2.1 mm) (Supplementary Figures S3, S4). Ignoring the height and shape of the liquid drop, then, the cross-sectional area of illumination represents ∼1.3% of the drop. Therefore, despite having higher irradiance when integrated with the microscope (28 W/cm^2^) than with direct exposure (15 W/cm^2^), the smaller exposure area results in much lower overall uncaging efficiency. Quantification of ADE released upon exposure shows that a starting concentration of 10 mM pcADE yields a nominal ADE concentration of only 2.5 mM, even after 5 s of UV exposure at maximum LED intensity. Compared to our previous PVM experiments by direct diffusion of ADE ligand (Ramakrishnan et al., 2021d), this concentration of ADE is not only four times lower, but is also achieved much more gradually over several seconds of exposure, as opposed to the expected µs to ms by diffusion. Kinetic analyses of PVM data recorded for three pcADE-soaked crystals of different sizes indicate that the apparent uncaged ADE concentration in crystal is even lower (discussed below).

**TABLE 2:** Percent ADE released at different UV (365 nm) LED exposure times.

	1	2	3	Mean
Direct exposure				
1 s	32.9	34.5	36.9	35 ± 2
5 s	61.0	57.8	66.9	62 ± 5
10 s	64.3	60.4	67.5	64 ± 4
Microscope-integrated^a^				
1 s	8.9	8.1	10.4	9.0 ± 1
5 s	23.7	28.1	24.5	25 ± 2
10 s	28.5	30.0	26.1	28 ± 2

a UV LED is mounted on the microscope and the light is focused through the microscope optics.

### 3.2 Comparing transition uniformity at different crystal positions

**FIGURE 2:**
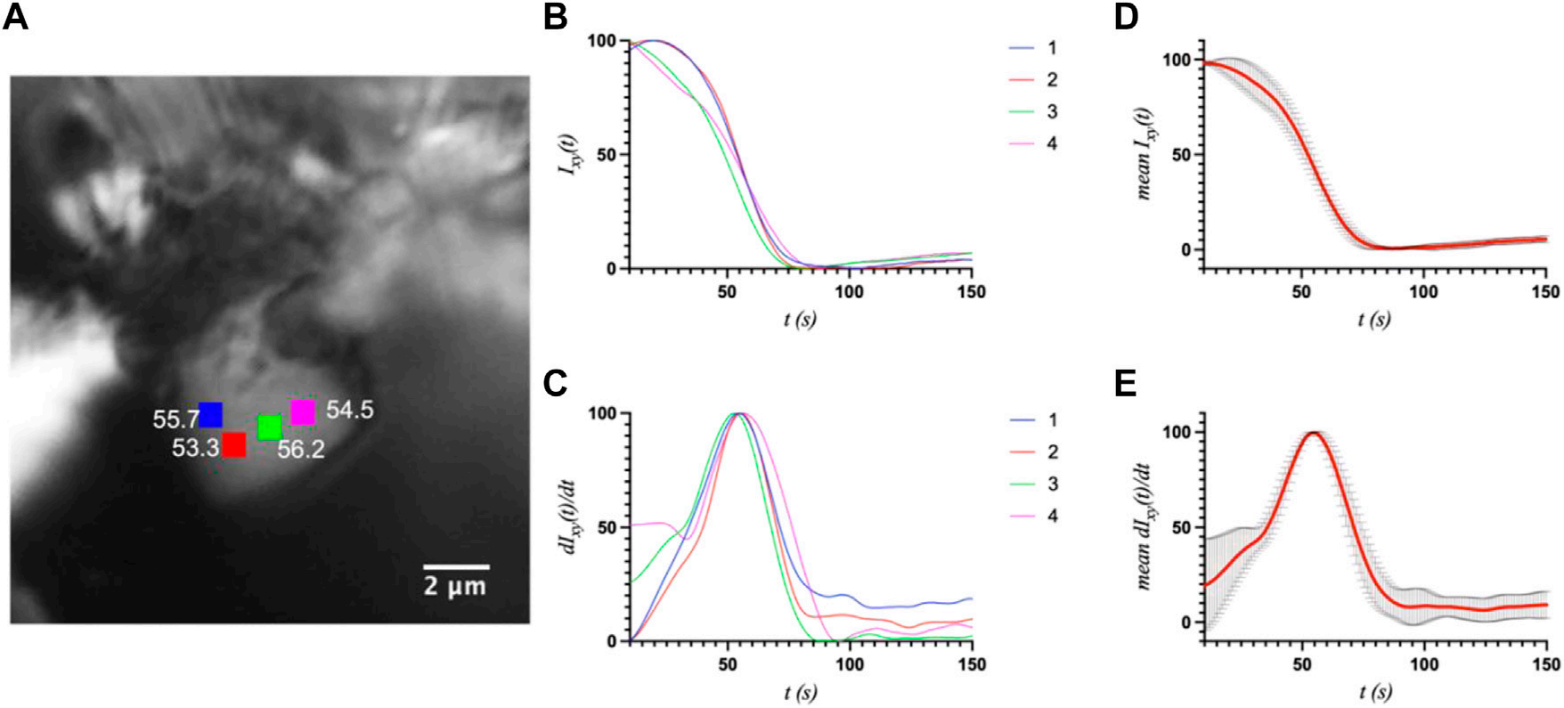
**(A)** The optical microscope image of small crystal S2 (5.3 × 6.0 μm^2^) with the measured ROIs and their respective T1 values (s) indicated. **(B)** and **(C)** The intensity and first derivative curves for the individual ROIs. **(D)** and **(E)** The average intensity and first-derivative curves for all ROIs, and their standard deviations.

**FIGURE 3:**
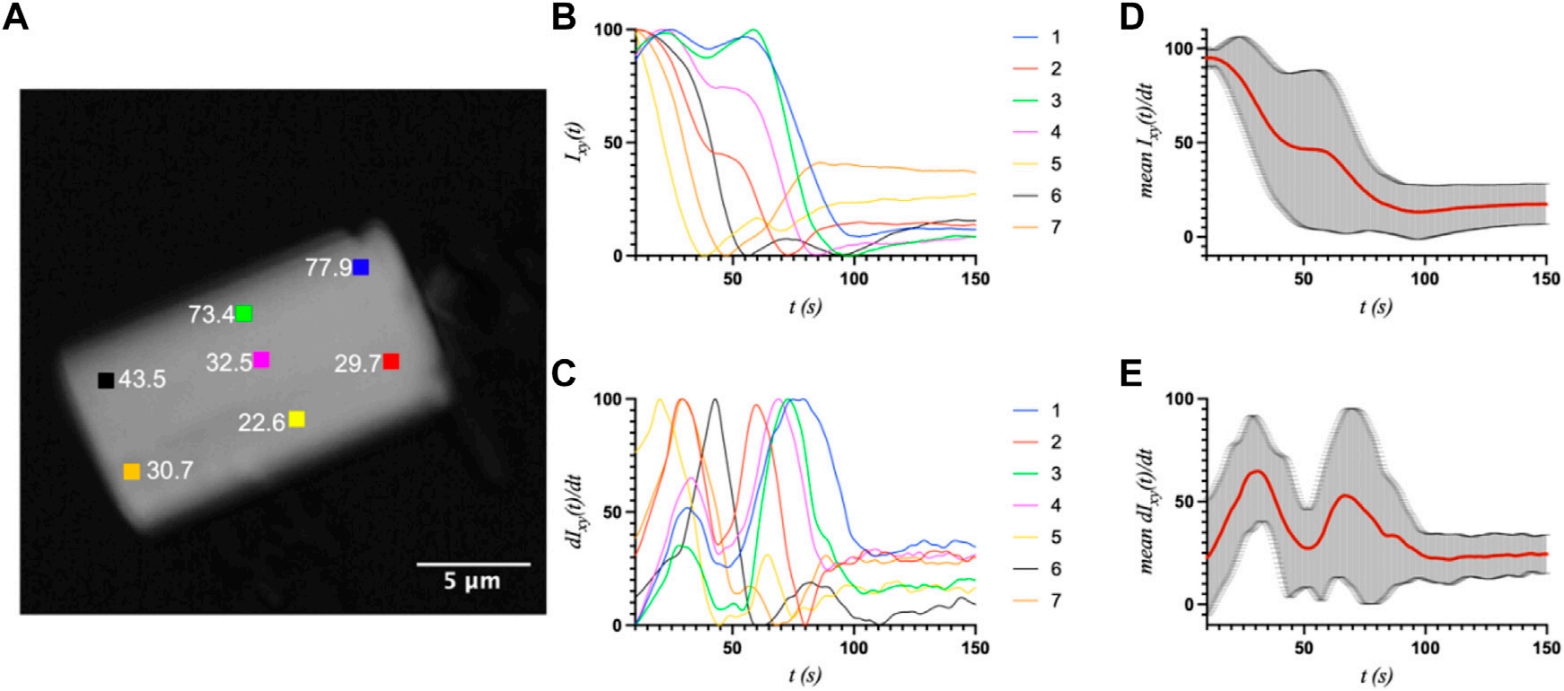
**(A)** The optical microscope image of medium crystal M3 (8.5 × 15.2 μm^2^) with the measured ROIs and their respective T1 values (s) indicated. **(B)** and **(C)** The intensity and first derivative curves for the individual ROIs. **(D)** and **(E)** The average intensity and first-derivative curves for all ROIs, and their standard deviations.

**FIGURE 4:**
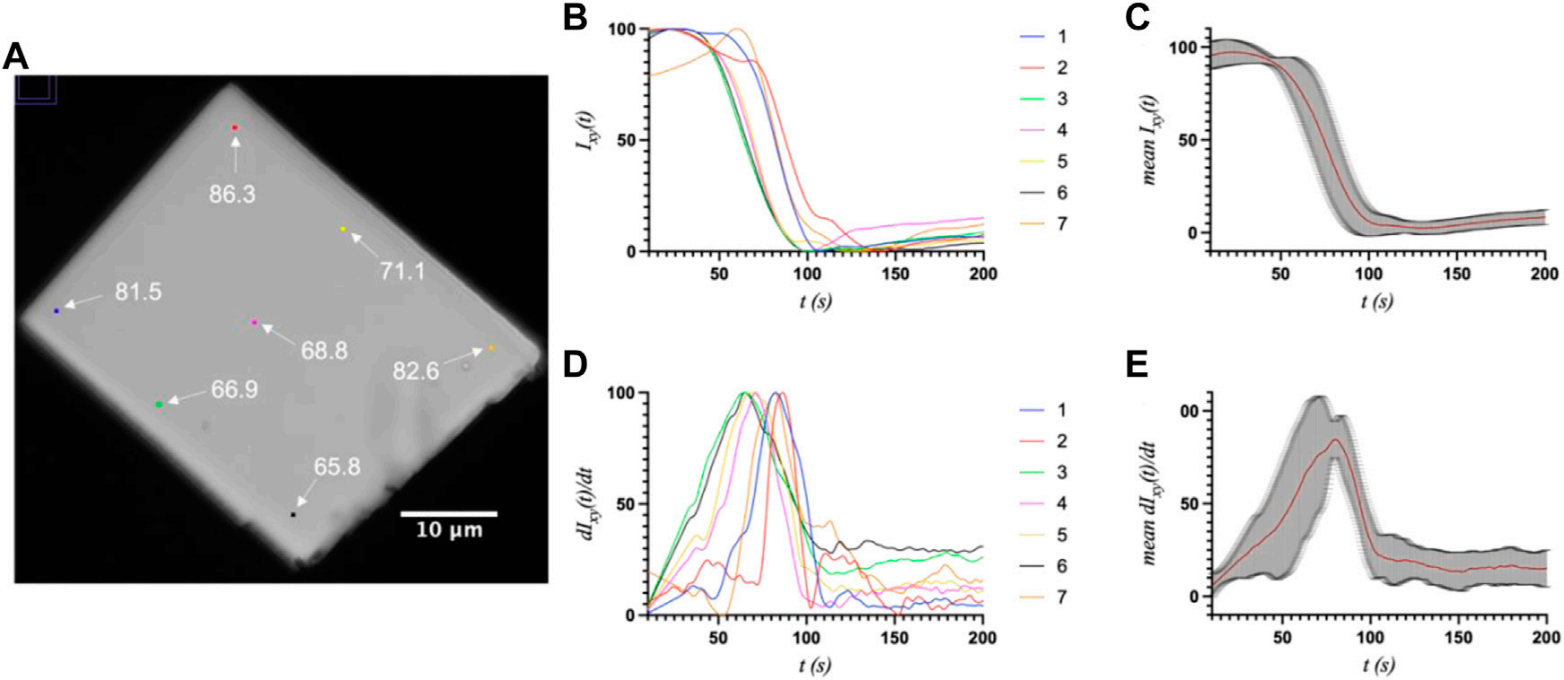
**(A)** The optical microscope image of large crystal L1 (33.0 × 42.2 μm^2^) with the measured ROIs and their respective T1 values (s) indicated. **(B)** and **(C)** The intensity and first derivative curves for the individual ROIs. **(D)** and **(E)** The average intensity and first-derivative curves for all ROIs, and their standard deviations.

First, a whole-crystal PVM analysis was performed, which provides a quantitative representation of what is observed in the PVM videos (Supplementary Material), in that the transition occurs in one or more waves that propagate throughout the crystal. For each crystal, up to seven regions of interest (ROI) (10 × 10 pixels^2^) were independently analyzed by plotting the average intensity over time. The selected ROIs were chosen in such a way as to best represent the transition for the entire crystal while also excluding crystal edges or where severe cracking was observed, as these areas showed more non-uniformity during imaging, particularly upon crystal motion. The position of the major peak in the first derivative of each intensity plot denotes the time of the transition (T1) for that particular ROI (Table 1). Figures 2–4 show the optical microscopy images of three crystals of different sizes (S2, M3, L1) as well as their intensity and first derivative curves. In general, the smaller crystals suffered most from motion throughout the transition, making data acquisition and analysis more difficult. Crystal S2, however, was immobilized on one edge by being clustered with other crystals, and exhibited mostly uniform and overlapping curves for the four selected ROIs (Figure 2). The mean T1 for crystal S2 was ∼54.9 ± 1.3 s (Table 1), indicating a slow but rather uniform transition. Crystals M3 (T1: 44.3 ± 22.3 s) (Figure 3) and L1 (T1: 74.8 ± 8.4 s) (Figure 4) reveal more variation in T1 as a function of position within the crystal. The data as a whole, however, indicate a weak relationship between the magnitude and variation of T1 with respect to crystal size. This is quite different from the previously reported experiments with direct mixing with ADE, which showed a much stronger correlation, where the mean times of transition and their ranges became noticeably larger as crystal size increased (Ramakrishnan et al., 2021d). This is understandable because the light-triggered transition is less dependent on the diffusion of ADE into the crystal, and is instead largely driven by the equilibrium between caged and uncaged ADE during and after UV exposure.

### 3.3 Comparing transition uniformity across different ROI sizes

**FIGURE 5:**
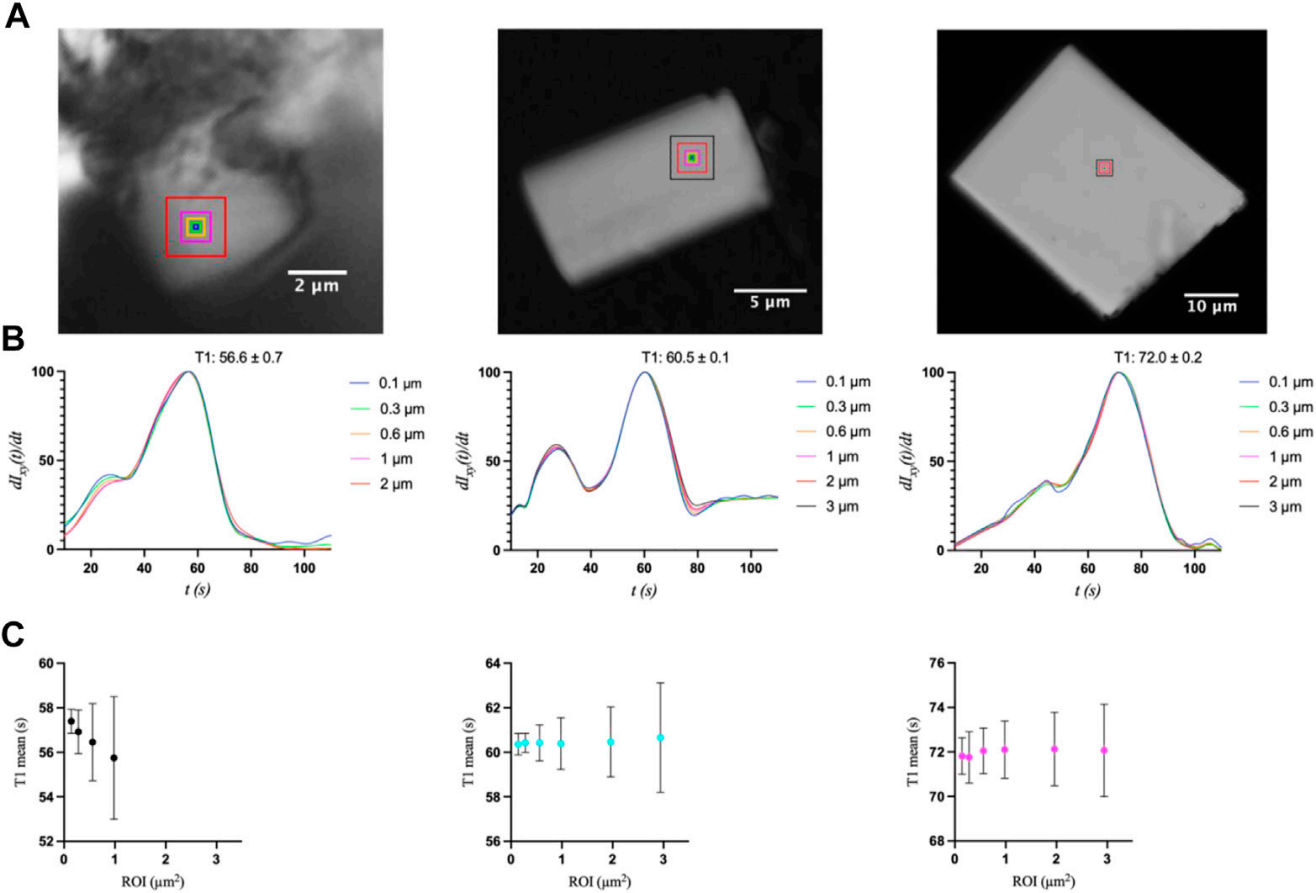
**(A)** Optical microscopy images of S2, M3, and L1 crystals with their respective ROIs (indicated as boxes) of increasing size with side-lengths of 0.1, 0.3, 0.6, and 1 μm, as well as 2 and 3 µm for crystals M3 and L1. **(B)** First-derivative curves and **(C)** mean T1 values with standard deviations for each ROI size.

A second and more informative analysis was used to investigate transition uniformity by comparing the peak positions at the pixel level for concentric ROIs of increasing size for the same three crystals (Figure 5). The idea behind this approach is that non-uniformity may increase with increasing ROI, and that the degree of variance may also increase as a function of crystal size, as was observed in the ligand mixing experiments (Ramakrishnan et al., 2021d). Whereas the first analysis probed the entire crystal by selecting representative positions, this analysis focuses on a specific area whose size (up to 3 µm) is most relevant to X-ray diffraction experiments with X-ray free electron lasers (XFELs). The ROIs here were squares with side-lengths of 2, 4, 8, 14, 28, or 42 pixels, corresponding to ∼0.1, 0.3, 0.6, 1, 2, and 3 μm, respectively (Figure 5). For S2, the ROIs of 2 and 3 µm were omitted from the analysis, as regions of this size could not be applied without including the section of the crystal that was part of the cluster of crystals, which exhibited different and inconsistent transitions.

**FIGURE 6:**
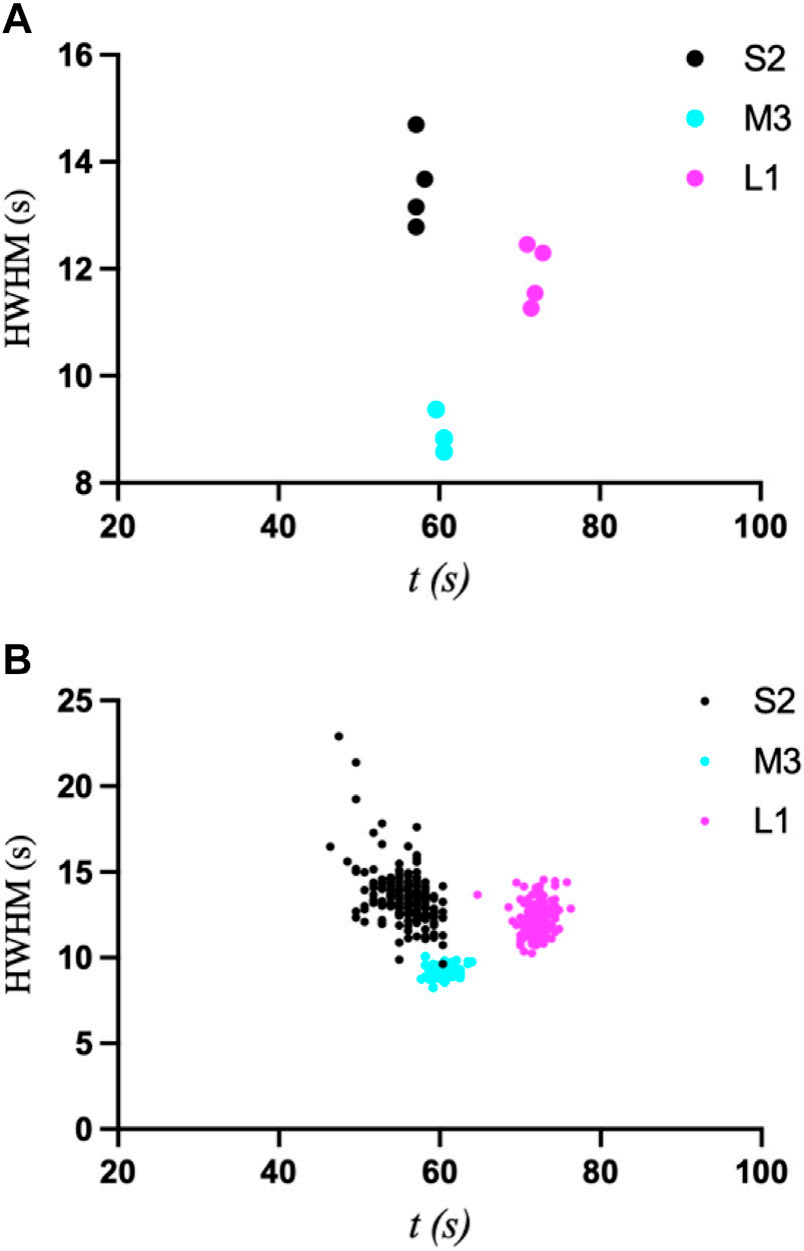
Plots of halfwidth at half-maximum (HWHM) vs. time for ROI sizes of 0.1 × 0.1 µm^2^ **(A)** and 1 × 1 µm^2^ **(B)** for crystals S2, M3, and L1, which compare their widths of transition.

The first interesting observation is that the first derivatives of the intensity curves all exhibit a smaller initial peak or shoulder that occurs before the primary transition peak (Figure 5B). That is, the start of the transition is gradual, and even slows down, followed by a more Gaussian-like transition. This behavior is indicative of the gradual release of ADE, whose binding kinetics are likely non-uniform throughout the UV exposure and in achieving equilibrium. Once T1 is reached, however, (55.7, 60.4, and 72.1 s for the 1 µm × 1 µm ROIs of S2, M3, and L1, respectively), the latter half of the transition peaks are much more consistent, with an expected Gaussian profile. The mean T1 values and their standard deviations were then compared as a function of ROI size using a *k-means* clustering algorithm (Figure 5C). For a more accurate comparison, the data for the primary peak only were used for this analysis. All three crystal sizes exhibited greater variation in T1 as ROI increased. These results are similar to those observed upon ADE mixing (Ramakrishnan et al., 2021d), as reflected in the comparison of T1 standard deviations between crystals of similar size in the two studies (Supplementary Table S1). The primary difference is that the smallest crystal (2.6 µm × 1.2 µm) analyzed with ADE mixing was shown to be independent of ROI size, exhibiting a mostly uniform transition throughout the entire crystal (Ramakrishnan et al., 2021d). However, this crystal was significantly smaller than crystal S2 (6.0 µm × 5.3 μm) in this study and is more comparable to the size of the medium crystal (6.7 µm × 3.7 μm) analyzed in the previous study. Unfortunately, smaller crystals were not measurable in the uncaging experiments as the energy release upon UV exposure and photolysis caused the crystals to move out of the field of view. We then compared the transition widths for ROIs of 0.1 µm × 0.1 μm and 1 μm × 1 μm by computing the half-width at half-maximum of the first-derivative peaks (Figure 6). From this analysis, it is clear that crystal M3 exhibited the shortest and most “uniform” transition window out of the three crystals. However, given the level of non-uniformity observed overall, as a result of gradual and insufficient release of ADE, the differences in the widths of transition may not be obvious with respect to crystal size.

### 3.4 Kinetic simulation of the phase transition using polarized video microscopy results

**FIGURE 7:**
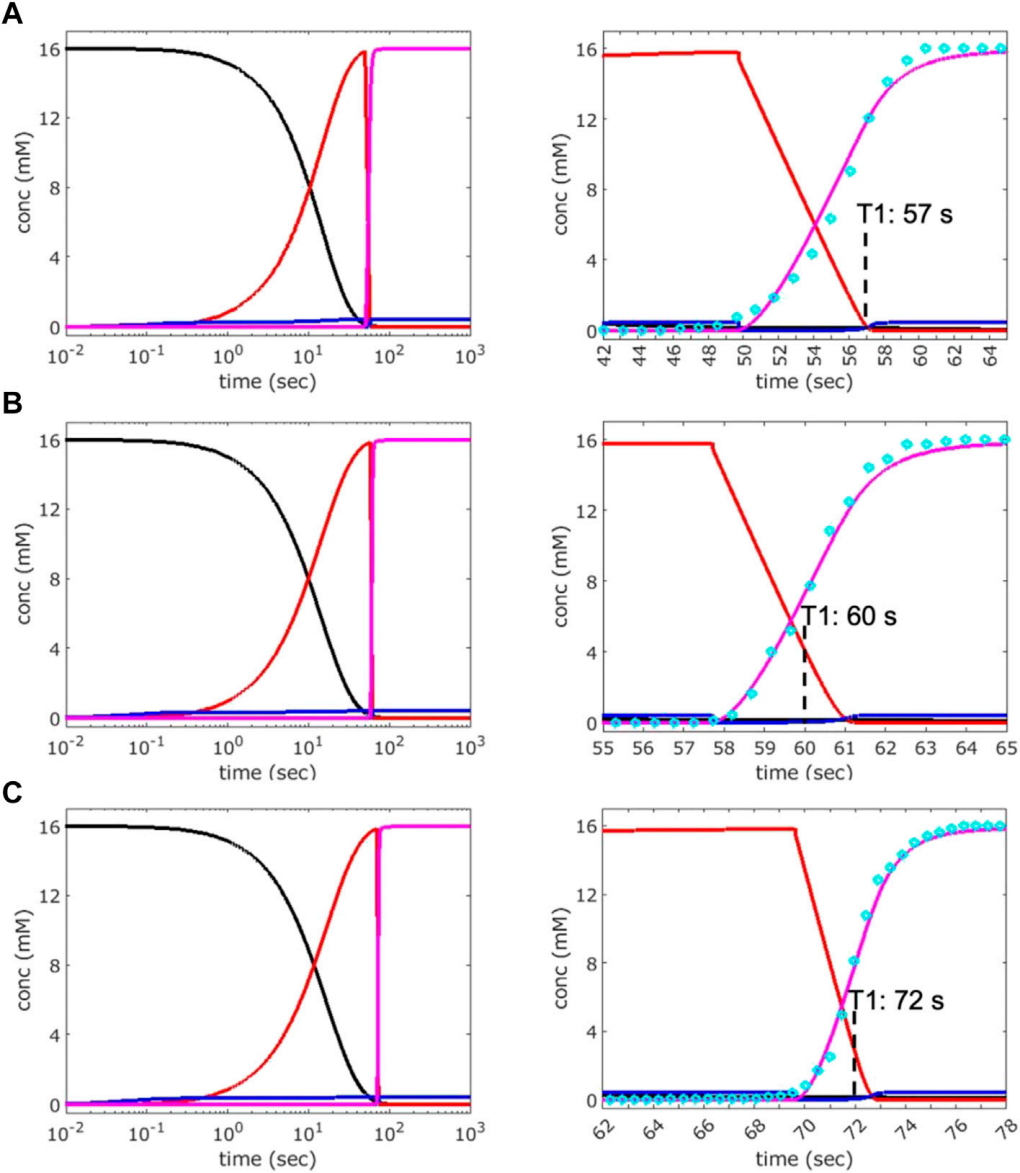
Analysis of the kinetics of transition observed by PVM. Kinetic simulations were performed by adjusting bulk adenine concentration (B0) and forward rate constants (k_*op*_, k_*on*_, and k_*f*_) to achieve the best fit to the transition curves (cyan circles) obtained by PVM for crystals S2 **(A)**, M3 **(B)**, and L1 **(C)**. The data used are from the 1 × 1 µm^2^ ROI described in Figures 5, 6. Left: plots showing the concentration of various species over time: apo1/apo2 (black), IB∙ade (red), B∙ade (magenta). Right: expanded views of the transition windows whose inflection points (magenta curve) represent the times T1. An RNA concentration of 16 mM was used for the simulations, which is the estimated concentration of each unique apo conformation (apo1, apo2) in crystal, calculated based on the unit-cell volume and two molecules of each apo conformer per unit cell.

To further understand the phase transition at the molecular level, the PVM data were used to fit kinetic simulations by applying our previously established four-state solution kinetics model (Eq. 1) (Stagno et al., 2017b; Ramakrishnan et al., 2021a; Ramakrishnan et al., 2021c). The conformational changes associated with ligand binding involve two distinct apo conformations (apo1/apo2), a ligand-bound intermediate (IB∙ade) and the converted (switched) ligand-bound conformation (B∙ade). As we are concerned only with the forward reaction in the SSPT in crystals, only the forward rate constants (k_*op*_, k_*on*_, and k_*f*_) are considered in this analysis. Using the 1x1 µm^2^ ROI PVM results for crystals S2, M3, and L1 (Figures 5, 6), kinetic simulations were performed to achieve a best fit to the transition data in each case (Figure 7; Table 3). Of note, a very narrow range (0.43–0.46 mM) of bulk ADE concentration (B0) was required for accurate fitting. This value is significantly lower than the nominal concentration of ADE (2.5 mM) released from a 10 mM pcADE drop after 5 s of exposure (Table 2). One obvious explanation is the effects of slow and gradual uncaging of pcADE and the subsequent non-homogeneous accumulation of ADE in the crystal that drives the SSPT. Another factor could be the uncertainty regarding the conversion of molecular species in the crystal environment as opposed to being free in solution. In particular, apo1 and apo2 in solution are in an equilibrium exchange with very different populations, whereas in crystal they are in equal population and restrained by lattice contacts. Given these factors, an observed (apparent) concentration of ∼0.45 mM is very reasonable. Moreover, the narrow range of this concentration among crystals of three different sizes with different transition profiles intrinsically validates this analysis. The k_*op*_ rate constants were also comparable: 2.35 × 10^−4^, 2.00 × 10^−4^, and 1.6 × 10^−4^ M^−1^s^−1^ for crystals S2, M3, and L1, respectively. The forward rate constant for the formation of IB∙ade, k_*on*_, was set to an optimized value of 3.00 × 10^−2^ M^−1^s^−1^ for all three crystals, as changes to this rate constant exhibited little effect on the kinetic profiles since the rate of accumulation of the ligand-bound intermediate is mostly dependent on B0 and therefore independent of crystal size. The final conformational switch to B∙ade, which is the rate-limiting step of the reaction and the most likely to be affected by crystal size, exhibited a rate constant (k_*f*_) of 0.5 M^−1^s^−1^ for S2, and very similar and somewhat slower values for M3 and L1 of 1.0, and 0.9 M^−1^s^−1^ respectively. Although slower due to the much lower ADE concentration, these rate constants and the simulated kinetics are consistent with previous results obtained for ADE mixing experiments (Ramakrishnan et al., 2021a; Ramakrishnan et al., 2021c). It is important to note that the uncaged ADE concentration in the crystals cannot be measured directly. Therefore, estimating the concentration by fitting to the solution kinetics model is the most reliable method to gain insight into the conformational phase transitions.

**TABLE 3:** Parameters used in the kinetic simulations for fitting to PVM data.

	S2	M3	L1
B0	0.46	0.42	0.43
k_*o*_	2.35E-04	2.00E-04	1.60E-04
k_*on*_	3.00E-02	3.00E-02	3.00E-02
k_*f*_	0.5	1	0.9

B0: Bulk adenine concentration (mM). k_*op*_, k_*on*_, and k_*f*_ : forward rate constants (M^−1^s^−1^).

## 4 Discussion

Time-resolved studies have been limited historically to systems involving only small and local conformational changes (mostly of photoreceptors). But the question still remains of whether TRX can be used routinely to study ligand-induced structural changes. The vast majority of cellular functions are driven by molecular interactions. A large subset of those interactions likely involves conformational changes that, in crystalline form, would be impeded by lattice restraints. A subset of those might be capable of overcoming those restraints in the form of an SSPT. If an SSPT were to occur, it would have two minimum requirements: First, the ability to overcome existing lattice restraints, and second, to do so in such a way that the lattice order is maintained in the process. We have shown previously that, for riboA, the smaller the crystal, the greater propensity for uniform transition, indicating that lattice disorder resulting from ligand-inducible changes might be mitigated to some extent using very small crystals (Ramakrishnan et al., 2021d). Having a greater surface-to-volume ratio allows diffusion rates that are at least comparable to the phase transition in the crystal, which in turn allows for better time resolution to capture reaction intermediates (Schmidt, 2013) as well as increased plasticity to accommodate those changes. In addition, smaller crystals are less likely to be plagued by crystal nonuniformities such as high mosaicity and lattice defects. However, TRX experiments using an XFEL revealed heterogeneous diffraction data, most likely owing to slow and non-uniform transition and variation from crystal to crystal (Stagno et al., 2017b; Ramakrishnan et al., 2021a). Here, we aimed to explore the feasibility of using a photocaged ligand to achieve a transition that is more uniform, and that may be less dependent on crystal size and shape, which are difficult to control during sample preparation for TRX experiments.

The PVM experiments using riboA crystals pre-soaked with 10 mM pcADE demonstrated that the SSPT is light-inducible in crystals of various sizes. However, use of pcADE presented several practical challenges. First, the compound was much less soluble in the crystal stabilization buffer compared to ADE, which is very soluble at >20 mM. Although 10 mM pcADE could be achieved in this buffer, it tended to crash out of solution over time, particularly in the 1 µl drop volumes, possibly due to rapid solvent evaporation from the large surface area relative to its volume and long UV exposures. Second, only a small fractional volume of the drop is exposed as a result of the beam diameter being much smaller than the diameter of the drop, resulting in gradual and incomplete uncaging and non-uniform saturation of ligand binding sites. For these reasons, and other possible contributing factors, such as heat exchange or heat-induced diffusion of caged and uncaged species, the pcADE under these experimental conditions and settings was shown to be insufficient for achieving rapid release of ADE at high enough concentrations. At maximum LED intensity (4.5 Amps) and very long exposure time (5 s), the highest percent yield that could be achieved was 25%, which amounted to a nominal ADE concentration of 2.5 mM.

The analysis of the transition curves revealed that, in general, the mean transition times and transition widths were less dependent on crystal size than those previously observed upon mixing with 10 mM ADE. This finding may be explained by the limited uncaging of pcADE as a result of poor uncaging efficiency and the small fractional volume that is exposed relative to the total drop volume. The gradual release of ADE and the potential changing equilibrium of uncaged and caged ligand inside the crystal explains the non-uniform transitions observed across the surfaces of riboA crystals. From a kinetics perspective, this can be described by the non-homogenous accumulation of the ligand-bound intermediate and the energy necessary to overcome lattice restraints. It is noteworthy to point out that the transition times and transition widths were challenging to measure accurately, and the observed values were affected unavoidably by several factors, such as crystal movement and ligand exchange between the inside and outside of a crystal. The crystal movements were particularly severe with small crystals. Moreover, the greater plasticity and sensitivity of smaller crystals, which are to be exploited for achieving transition uniformity under rapid ligand-saturating conditions, may also make these crystals more susceptible to variation under non-ideal conditions, such as temperature changes and non-uniform ligand saturation that result from poor uncaging efficiency. Nevertheless, the transition profiles could be successfully and reasonably described through a simulated kinetic fitting.

These results provide fundamental insight into the use of photocaged ligands for TRX studies of large conformational changes involving phase transitions. However, it is essential that the practical limitations of ligand solubility and uncaging efficiency are addressed. One way to help mitigate this problem is the synthesis of a different cage with a hydrophilic group attached to improve solubility. On the other hand, pump-probe XFEL experiments using a nanosecond UV laser have the potential to circumvent both of these problems. A sealed reservoir containing a slurry of microcrystals saturated in pcADE-containing buffer is far less susceptible to evaporation and ligand precipitation than is a 1 µl drop. As for uncaging efficiency, sample injections can be performed using capillaries with inner diameters less than 100 μm, and compact tunable lasers systems (e.g., Opolette, Opotek) can be implemented to achieve a desired spot size and power density, with live feedback from X-ray diffraction. The intrinsic dynamics of large RNAs with complex tertiary folds are directly correlated with structure-function relationships and conformationally driven mechanisms. Besides adenine riboswitch, we have illustrated the significant conformational changes that occur upon ligand binding for the flavin mononucleotide (FMN)- and tetrahydrofolate (THF)-binding riboswitches (Wilt et al., 2020; Wilt et al., 2021). However, single snapshots of apo and holo states, although highly informative, are still insufficient for achieving a complete understanding of the dynamics of conformational switching and how they correlate with function. The use of photocaged ligands and time-resolved crystallographic studies hold great potential for the field of RNA structural dynamics.

## Data Availability

Time-lapse microscopy videos for the crystals presented in this manuscript are available in the Supplementary Materials.
