# Supplementary figures and images for: Developing methods to study conformational changes in RNA crystals using a photocaged ligand
Source: Front Mol Biosci. 2022 Aug 16;9:964595. doi: 10.3389/fmolb.2022.964595 (PMC9424638; doi:10.3389/fmolb.2022.964595)

## Slide 1
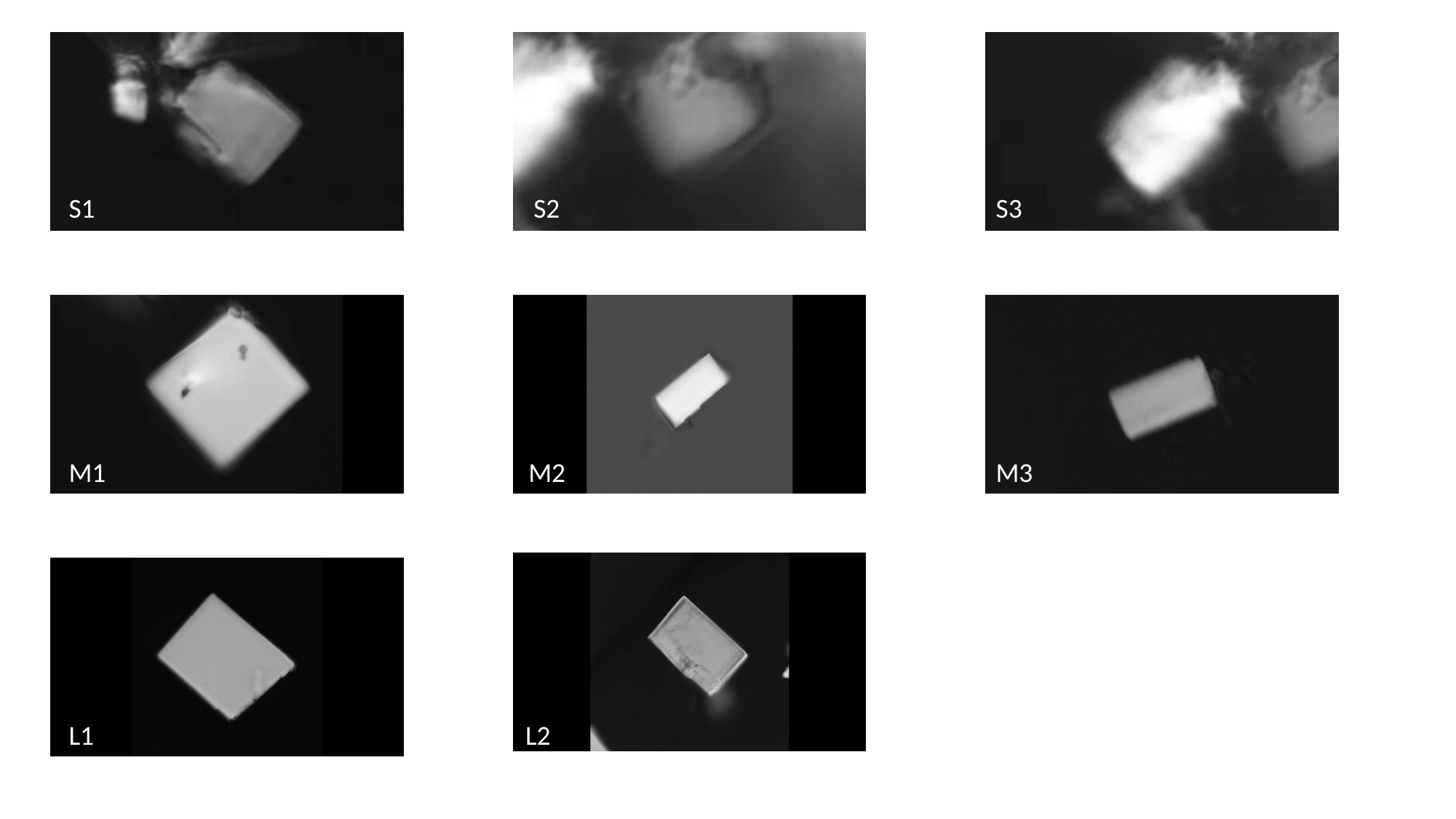

S2
S3
S1
M1
M2
M3
L1
L2

Supplement: Supplementary file 1 [file Presentation1.PPTX]
