# Supplementary material for: Developing methods to study conformational changes in RNA crystals using a photocaged ligand
Source: Front Mol Biosci. 2022 Aug 16;9:964595. doi: 10.3389/fmolb.2022.964595 (PMC9424638; doi:10.3389/fmolb.2022.964595)
Supplement: Supplementary file 2 [file DataSheet1.PDF]

Supplementary Material

1 Supplementary Figures and Tables

1.1 Supplementary Figures

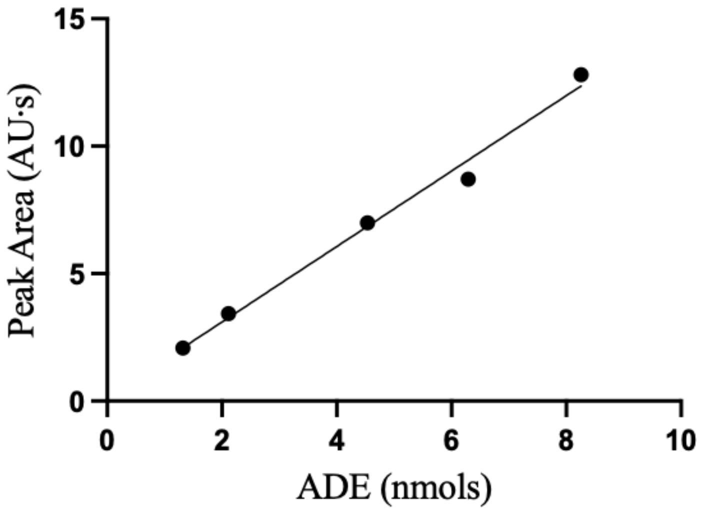

Supplemental Figure S1. Standard curve of HPLC elution-peak area vs quantity of ADE (R^2 = 0.9892). To best quantify the amount of ADE being released upon UV exposure, we first measured the elution-peak areas for different amounts of ADE (1.3, 2.2, 4.5, 6.3, 8.3 nmol) injected onto the column, whose concentrations were first determined by UV absorbance using a spectrophotometer.

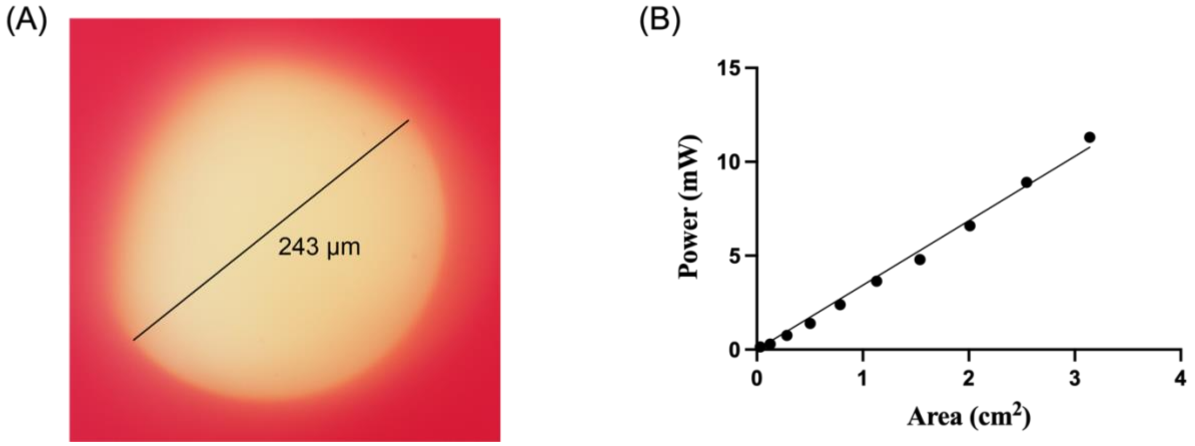

Supplemental Figure S2. (A) Diameter of the UV LED beam coming through the x100/1.45 Plan Apo Lambda objective. The diameter of the UV beam illuminating the sample in the PVM experiments was determined by exposing a slide colored with permanent marker for 2 min at 4.5 Amps. The photobleached area shows a mostly uniform beam with a diameter of ~243 µm, determined from an image taken with a 20x/0.75 Plan Apo objective, with a calibration of 2.70 pixels/µm. **(B)** Plot of power (mW) measurement as a function of field diaphragm area (R^2= 0.9930), illustrating uniformity of the beam through the microscope objective.

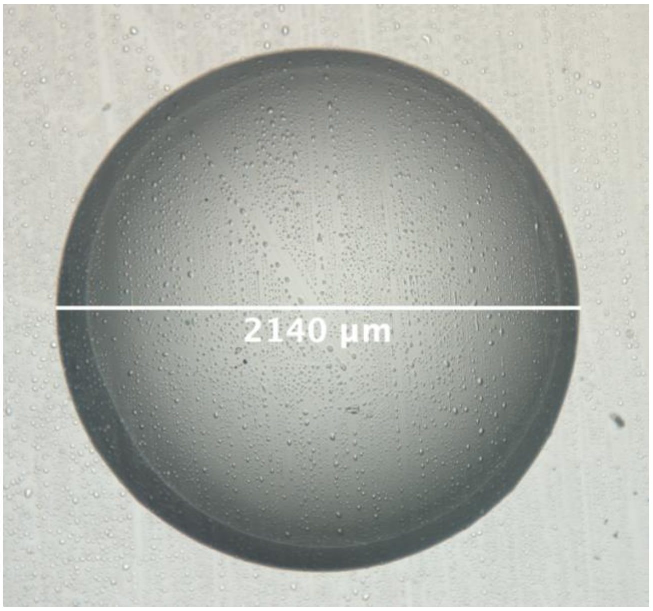

Supplemental Figure S3. Diameter of a 1 µL drop of stabilization buffer dispensed on the glass bottom dish. The image was taken using a Zeiss Axio Zoom V16 microscope with a Plan-NEOFLUAR Z 1.0x/0.25 FWD 56 mm objective. The diameter of the drop (~2.1 mm) was determined using ImageJ based on calibration of 0.76 pixels/µm.

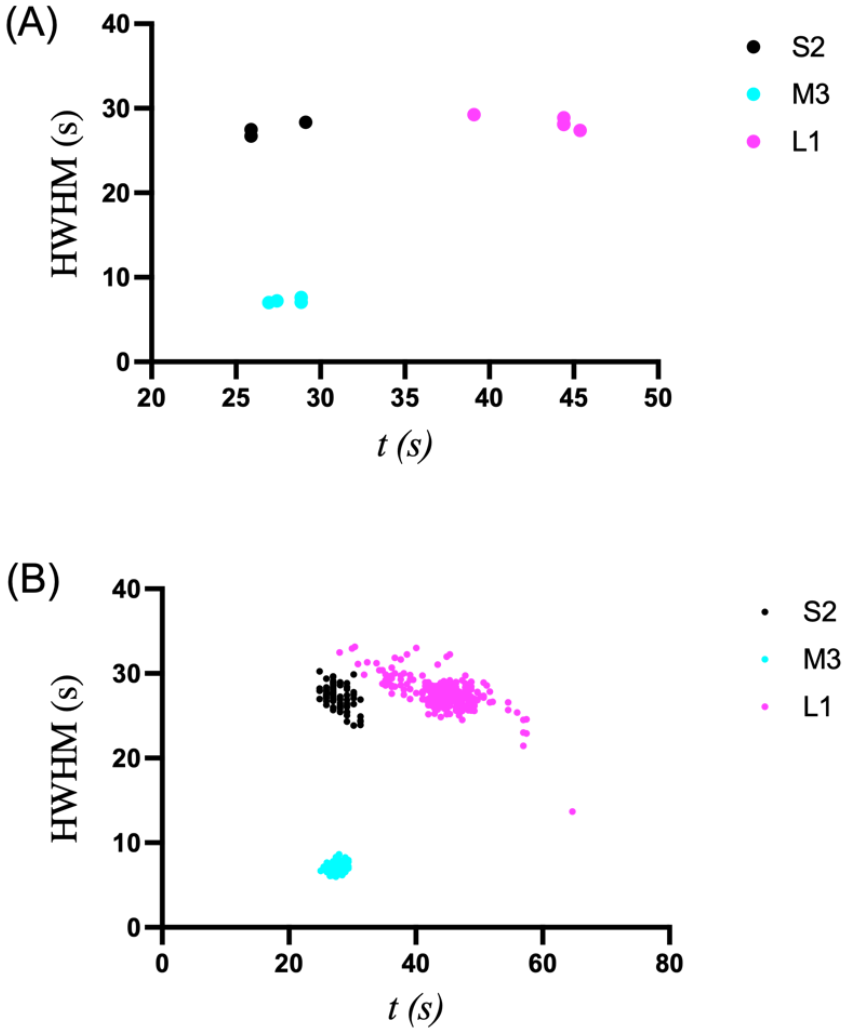

Supplemental Figure S4. Plots of half-width at half-maximum (HWHM) vs time for ROI sizes of 0.1×0.1 μm^2 **(A)** and 1×1 μm^2 **(B)** for crystals S2, M3, and L1, comparing their widths of transition. This plot shows the HWHM of the shoulder peaks present in their respective derivative curves (see Figure 5b).

1.2 Supplementary Tables

Supplementary Table 1. Mean T1 and standard deviations for three crystals of different sizes measured for phase transitions induced by direct ligand mixing (Ramakrishnan et al., 2021) as compared to photo-uncaging (this work).

		Mixing		Uncaging	
	ROI (μm)	mean T1	SD	mean T1	SD
**Small**	0.1	13.7	0.1	57.4	0.5
	0.3	13.7	0.1	56.9	1
	0.6	13.7	0.1	56.5	1.7
	1	13.6	0.2	55.7	2.8
**Medium**	0.1	15	0.1	60.4	0.5
	0.3	15.1	0.2	60.4	0.4
	0.6	15.1	0.3	60.4	0.8
	1	15.3	0.7	60.4	1.2
	2	15.7	1.5	60.5	1.6
	3			60.7	2.5
**Large**	0.1	33.1	0.2	71.8	0.8
	0.3	33.2	0.2	71.8	1.2
	0.6	33.1	0.4	72	1
	1	33	0.8	72.1	1.3
	2	32.7	1.5	72.4	1.8
	3			72.3	2.2

RAMAKRISHNAN, S., STAGNO, J. R., MAGIDSON, V., HEINZ, W. F. & WANG, Y. X. 2021. Dependence of phase transition uniformity on crystal sizes characterized using birefringence. *Struct Dyn,* 8**,** 034301.
